# Sociodemographic and Clinical Predictors of the Length of Psychiatric Inpatient Stay of Immigrants in Switzerland

**DOI:** 10.3389/fpsyt.2020.585798

**Published:** 2020-12-09

**Authors:** Renée Frizi, Barbara Lay, Erich Seifritz, Wolfram Kawohl, Benedikt Habermeyer, Patrik Roser

**Affiliations:** ^1^Department of Psychiatry and Psychotherapy, Psychiatric Services Aargau, Academic Teaching Hospital of the University of Zurich, Windisch, Switzerland; ^2^Department of Psychiatry, Psychotherapy and Psychosomatics, Psychiatric University Hospital, University of Zurich, Zurich, Switzerland

**Keywords:** immigrants, mental disorders, psychiatric hospitalization, inpatient treatment, length of stay

## Abstract

Immigrants with mental disorders have consistently been reported to spend shorter time in the psychiatric hospital compared to native patients. The aim of this study was to identify sociodemographic, clinical and migration-related predictors of a shorter length of psychiatric inpatient stay among immigrants in Switzerland. All patients of a foreign nationality admitted for inpatient treatment in the year 2016 (*N* = 279) were included in this study. The sample characteristics were drawn from the register of the psychiatric hospital. Within this sample, self-harm and substance use predicted a shorter inpatient treatment episode whereas disturbances of general psychosocial functioning were a predictor of a longer length of stay. As similar results were also reported for non-immigrant patients, the impact of these specific behavioral and social problems on the length of inpatient stay does not appear to be migrant-specific. Moreover, a country of origin outside Europe was a strong predictor of shorter length of stay pointing to inequalities of inpatient psychiatric treatment within the group of immigrants. Therefore, the cultural background and migrant history of immigrants in psychiatry need stronger consideration in order to eliminate disadvantages in mental health care.

## Introduction

Migration is a universal phenomenon across the whole globe and throughout the entire history of mankind. It has significantly increased during the ongoing globalization process with its complex social, economic and environmental consequences (1). A number of studies from Europe and North America has linked migration to psychological stress that may lead to manifest psychiatric symptoms and mental illness, particularly schizophrenia, alcohol and drug abuse, anxiety and depression as well as suicidal behavior (2). Interpretation of these findings should, however, consider that the process of migration is highly heterogeneous and its impact on mental health shows a large variation depending on the nature and reasons for the migration and the degree of acculturation (3). Of note, these factors are mainly influenced by the immigrant's personal and social background; the social, cultural, political, and economic conditions of the immigrant's home country; and the recipient society's attitude toward immigrants (3).

Despite the higher risk of developing mental disorders, immigrants from all regions of the world show a general tendency toward a lower use of mental health care services, particularly of specialized mental health and hospital care, compared to the native populations in Europe as well as in North America (4–7). However, there are significant differences within the group of immigrants with the lowest rate of mental health care use by immigrants from East Asia and Latin America and the highest rate among immigrants from Middle East and North Africa (4, 6). Moreover, mental health services are underutilized among recent and labor immigrants, respectively, compared to long-term residents, undocumented immigrants or refugees (6). Apart from the lower utilization, immigrants are also less likely to be referred to psychiatric consultation when presenting psychiatric symptoms (8) and, once they have accessed the mental health care service, also show a higher rate of treatment dropout, compared to the native population (9). Besides these individual factors, structural barriers for the access to mental health care facilities including lack of insurance and language barriers could be identified as a relevant reason for lower rates of mental health care use as well as higher rates of discontinuation of psychiatric outpatient treatment among immigrants (5).

With regard to psychiatric inpatient treatment, immigrants appear to be at a significant disadvantage as well. A large Swiss register study showed that immigrants have lower rates of psychiatric hospitalisations, have more emergency and compulsory admissions, experience more physical restraint, spend shorter time in hospital, are admitted with lower illness severity, and are more likely not to be readmitted, compared to native patients (10, 11). These results particularly applied to immigrants from South Europe, former Yugoslavia, Turkey, East Europe and more distant countries outside Europe, but not to patients from West and North Europe. A further analysis of the data focussing on gender-specific utilization of psychiatric services in Switzerland revealed that mainly male immigrants from Turkey, East Europe and more distant countries showed higher admission rates compared to females from the same countries, pointing to inequalities in mental health care use among immigrants with different migration background (12). Regarding the psychiatric diagnoses, the prevalence of psychotic disorders was, interestingly, similar in immigrants and Swiss patients, whereas the rate of neurotic, stress-related and somatoform disorders was two to three times higher among immigrants from former Yugoslavia, Turkey and more distant countries, and the rate of personality and behavior disorders was three times lower among immigrants from East Europe, compared to natives (13).

A recently published study by our research group investigated the relationship between sociodemographic and clinical variables and the length of inpatient stay in a Swiss psychiatric hospital (14). In the study sample of 1'479 patients that were admitted in the year 2016, several predictors of the length of stay were identified. Among these, of note, immigration, as defined by foreign nationality, was strongly associated with a shorter length of stay compared to Swiss patients. It is, however, not clear whether this result can be explained by sociodemographic, clinical and/or migration-specific factors. We therefore performed a further analysis on the subsample of immigrants (*N* = 279). Specifically, we aimed to analyse more thoroughly migration-specific patient characteristics that were not considered in the former study. Moreover, we expanded the analysis including clinical symptoms and social functioning of these patients as assessed by the Health of the Nations Outcome Scales (HoNOS) which rarely have been evaluated in immigrants in previous studies. The aim of this study was (A) to assess the sociodemographic, clinical, and migration-specific characteristics of immigrants in psychiatric inpatient care, (B) to explore the association between patient characteristics and length of stay, (C) to identify predictors of length of stay, and (D) to draw conclusions about the specific needs of immigrants on a mental health care level.

## Methods

### Study Sample

The Department of Psychiatry and Psychotherapy of the Psychiatric Services Aargau (PDAG) provides inpatient mental health care for the Swiss Canton of Aargau, a catchment area of about 680'000 people with mainly suburban (> 10'000 inhabitants) and rural (< 10'000 inhabitants) communities located between the metropolitan areas of Zurich, Bern and Basel [for detailed information on the catchment area, see Stulz et al. (15)]. Moreover, it is the only psychiatric facility for acute inpatient care in duty to provide treatment for all compulsorily admitted people in the Canton of Aargau.

According to the national legal requirements, all psychiatric hospitals in Switzerland are obligated to record all admissions and discharges within their catchment area and to annually report detailed information including sociodemographic, diagnostic and treatment-related characteristics in a standardized form to the Federal Office of Statistics. The hospital doctors in charge of the respective patient are responsible for the documentation. The completion of forms and consistency of information are regularly monitored. Therefore, data on the inpatient episodes are almost complete and can be regarded as sufficiently reliable.

For this study, all patients between 18 and 65 years admitted to the Department of Psychiatry and Psychotherapy of the PDAG between 1 January 2016 and 31 December 2016 were included. In the case of multiple admissions of one patient in this given time period, only the first inpatient episode was considered.

In the year 2016, a total of 1'607 patients were admitted to the Department of Psychiatry and Psychotherapy of the PDAG. Of these, 128 patients were excluded because of missing relevant data. Of the remaining sample of 1'479 patients, a total of 279 patients (18.9%) were immigrants. According to the psychiatric register, the term “immigrant” was defined as any person of a foreign nationality, including refugees, short-term immigrants and long-term residents who were not born or naturalized in Switzerland.

### Study Measures

The patients' sociodemographic and clinical data were drawn from the psychiatric register stored on the electronic medical database of the PDAG.

The standard sociodemographic characteristics included information on age, gender, marital status and employment. Moreover, migration-specific variables were assessed, including the period of residence in Switzerland (≤ 5, 5–10, 10–20, 20–30, 30–40, > 40 years), country of origin, legal residency status (documented vs. undocumented immigration), language barriers and migration generation (first generation vs. second generation). Given the relatively small sample size, the countries of origin were grouped into three categories: immigrants coming from a Swiss neighbor country (i.e., Austria, France, Germany, and Italy), from another European country or from a non-European country. Language barriers were estimated by the psychiatrists according to the degree of difficulties in verbal communication.

The clinical variables include the principal psychiatric diagnosis, based on ICD-10 diagnostic criteria, number of previous admissions, type of admission (voluntary vs. compulsory), HoNOS scores at admission and length of inpatient stay. The HoNOS (16) is an established rating instrument for the assessment of the severity of mental disorders and social problems. It consists of 12 items measuring four broader categories: behavior, impairment, symptoms, and social functioning. Each item is rated from 0 (no problems) to 4 (severe to very severe problems), resulting in a total score ranging between 0 and 48. According to the thresholds suggested by Parabiaghi et al. (17), total HoNOS scores higher than 13 are considered to indicate a high level of illness severity while lower scores indicate a rather moderate or low level of illness severity. The HoNOS ratings were made within the first 3 days after admission to the hospital by the doctors responsible for the treatment of the patients.

### Statistical Analysis

Length of hospital stay (LOS) was measured in days. The frequency distribution of LOS usually does not follow a normal density function; also in the present sample, it was highly skewed. We therefore naturally logarithmized this measure and used the transformed variable (LN_LOS) as the dependent variable in further statistical analyses [mean 2.82; standard deviation (SD) = 1.11]. Only for descriptive information we refer to the non-transformed LOS data (Table 1, Supplementary Table S1).

**Table 1:** Characteristics of the study sample (*N* = 279).

	**N of patients**	**%**	**Length of stay (days)**		
			**Mean**	**SD**	**Median**
**Sociodemographic variables**					
Gender					
female	123	44.1	29.1	22.9	26.0
male	156	55.9	23.9	19.5	20.0
Marital status					
single	112	40.1	26.6	23.5	21.0
married	98	35.1	27.1	21.6	20.5
separated, divorced, widowed	69	24.7	24.3	16.2	21.0
Employment					
employed	92	33.0	28.5	23.0	27.0
unemployed	186	66.7	25.1	20.3	20.0
**Migration-specific variables**					
Period of stay in Switzerland					
≤ 5 years	37	14.4	17.2	16.5	14.0
5-10	30	11.7	27.1	28.7	17.5
10-20	62	24.1	26.6	17.3	21.0
20-30	59	21.1	26.4	20.7	21.0
30-40	35	12.5	29.6	20.7	28.0
> 40	34	12.2	34.2	21.1	31.0
Country of origin					
neighbor country	110	39.4	29.4	20.4	28.0
other European country	123	44.1	26.9	21.5	20.0
non-European country	46	16.5	16.8	20.0	11.0
Legal residency status					
documented	255	91.4	26.7	20.9	21.0
undocumented / refugee	24	8.6	21.3	23.8	12.0
Barrier of language					
no	209	74.9	27.5	21.3	23.0
yes	70	25.1	22.3	20.7	16.0
Migration generation					
first	36	12.9	26.4	19.0	24.5
second	243	87.1	26.2	21.5	20.0
**Clinical variables**					
N of previous admissions					
0	99	35.5	25.6	18.6	21.0
≥ 1	162	58.1	25.8	22.4	20.0
Admission					
voluntary	197	70.6	28.6	20.9	25.0
compulsory	82	29.4	20.5	20.9	14.0
Principal diagnosis (ICD-10)					
F1 (substance use disorders)	61	21.9	21.2	16.6	18.0
F2 (psychotic disorders)	67	24.0	30.6	25.6	26.0
F3 (affective disorders)	75	26.9	31.1	19.8	30.0
other	76	27.2	21.6	20.0	16.5
Discharge					
regular	208	74.6	29.3	22.1	26.0
against medical advice	71	25.4	17.2	15.0	14.0
Length of inpatient stay (days)	279	100	26.2	21.2	21.0

*Note: Categories do not sum up to 100% in some variables due to missing values*.

*SD, standard deviation*.

Inspection of the HoNOS data at admission revealed missing data in single items. None of the cases, however, had a HoNOS rating with missing data in more than three items. We calculated the HoNOS total score adding up the scores of the 12 items which provides a total score ranging from 0 to 48. For computation of the HoNOS total score, we imputed missing values: missing item values were substituted with the mean score of all completed items.

We performed regression analyses to explore the kind of association between patient characteristics assessed at admission and the length of hospital stay (LN_LOS). As independent variables, we considered the sociodemographic, migrant-specific, and clinical patient characteristics given in Table 2. All variables were first examined separately using univariate regression analysis in order to explore the extent to which they are associated with the outcome.

**Table 2:** Association between sample characteristics and length of inpatient stay.

	**B**	**Beta**	**95% CI**	***P*-value**
**Sociodemographic variables**				
Age (years)	0.01	0.10	−0.00; 0.02	0.09
Gender, male	−0.27	−0.12	−0.53; −0.01	0.04
(*ref* female)				
Employment, unemployed	−0.09	−0.04	−0.37; 0.19	0.54
(*ref* employed)				
**Migration-specific variables**				
Period of stay in Switzerland (years)	0.17	0.26	0.09; 0.25	<0.001
Country of origin				
Neighbor country	0.36	0.16	0.09; 0.62	0.009
Other European country	0.11	0.05	−0.16; 0.37	0.432
Non-European country	−0.81	−0.27	−1.15; −0.47	<0.001
Legal residency status, undocumented/refugee	−0.39	−0.10	−0.85; 0.08	0.10
(*ref* documented)				
Barrier of language, yes	−0.27	−0.10	−0.57; 0.03	0.08
(*ref* no)				
Migration generation, second	−0.05	−0.01	−0.44; 0.34	0.80
(*ref* first)				
**Clinical variables**				
N of previous admissions, ≥ 1	−0.08	−0.03	−0.36; 0.21	0.60
(*ref* 0)				
Admission, compulsory	−0.59	−0.24	−0.87; −0.31	<0.001
(*ref* voluntary)				
Principal diagnosis (ICD-10)				
F1 (substance use disorders)	−0.21	−0.08	−0.53; 0.10	0.19
F2 (psychotic disorders)	0.18	0.07	−0.12; 0.49	0.25
F3 (affective disorders)	0.40	0.16	0.11; 0.69	0.007
Other	−0.38	−0.15	−0.67; −0.09	0.01
**HoNOS items**				
HoNOS 01, Aggression and overactivity	−0.17	−0.20	−0.27; −0.07	0.001
HoNOS 02, Self-harm	−0.11	−0.12	−0.23; −0.00	0.05
HoNOS 03, Substance use	−0.17	−0.24	−0.26; −0.09	<0.001
HoNOS 04, Cognition	0.14	0.14	0.02; 0.25	0.02
HoNOS 05, Physical health	0.14	0.16	0.04; 0.24	0.007
HoNOS 06, Hallucinations and delusions	−0.001	−0.001	−0.10; 0.10	0.98
HoNOS 07, Depression	0.19	0.20	0.08; 0.29	0.001
HoNOS 08, Other symptoms	0.03	0.03	−0.07; 0.12	0.59
HoNOS 09, Social relations	0.004	0.005	−0.10; 0.11	0.93
HoNOS 10, General functioning	0.29	0.35	0.19; 0.38	<0.001
HoNOS 11, Housing	0.08	0.10	−0.02; 0.18	0.13
HoNOS 12, Activities	0.10	0.12	0.00; 0.19	0.05
HoNOS, Total score	0.02	0.10	−0.002; 0.03	0.08

*Univariate regression analyses*.

*HoNOS, Health of the Nations Outcome Scales: up to 23 (8.2%) missing values in some items*.

*Length of stay (log-transformed)*.

In a second step, the effects of the explanatory variables were jointly estimated to take into account variable interdependence. We fitted a multiple regression model using forward stepwise variable selection. Probability of F-to-center was fixed at *P* < 0.05. In this analysis, we considered all sociodemographic, migrant-specific and clinical variables for which a significant effect may be assumed (*P-*value of < 0.1 in the bivariate analysis). Seeing that psychiatric diagnoses are strongly overlapping with symptomatic and behavioral problems assessed by the HoNOS, and likewise, the HoNOS total score cannot be regarded as independent from its constituents (HoNOS single items), we did not consider “psychiatric diagnoses” and “HoNOS total score” in this model in order to avoid statistical problems of multicollinearity, but used the HoNOS single items instead.

Data were analyzed using IBM SPSS Statistics for Windows, Version 25.0 (Armonk, NY: IBM Corp.).

## Results

### Sociodemographic and Clinical Characteristics

The sociodemographic and clinical sample characteristics are given in Table 1. The mean age of immigrants (*N* = 279) was 40.5 years (SD = 12.1). Most immigrants had a legal residence permit, belonged to the second generation and had no relevant language barrier. Less than one tenth of this sample were refugees. Half of the immigrants have stayed in Switzerland for more than 20 years and the majority of them came from countries within Europe. Whereas, the 279 inpatient treated immigrants included in this study are representing 18.9% of the total sample of 1'479 inpatients, the percentage of immigrants in the general population of the Canton of Aargau in 2016 was 24.5% (18). This suggests that immigrants are underrepresented in psychiatric inpatient treatment. With respect to clinical characteristics, most immigrants had at least one previous inpatient episode and were admitted on a voluntary basis. The most frequent psychiatric diagnoses included substance use, psychotic and mood disorders. The mean HoNOS total score of 17.2 (SD = 7.0) indicates that the patients were admitted to inpatient treatment with a rather high level of illness severity. In Supplementary Table S1, the statistics for the 12 HoNOS scales are detailed. The rate of one fourth of the immigrants that were discharged against medical advice is very comparable with native patients (10). The mean length of stay was 26.2 days (SD = 21.2).

### Association Between Sample Characteristics and Length of Stay

Further analysis of the data using univariate regression analysis revealed that distinct sociodemographic and clinical parameters, particularly gender, type of admission, psychiatric diagnosis and the HoNOS at admission, were significantly associated with the length of inpatient stay (Table 2). In detail, male patients, patients with compulsory admission and patients with aggressive and overactive behavior, self-harm as well as substance use, according to the HoNOS, spent shorter time in hospital. On the other hand, affective disorders as well as cognitive problems, physical illness or disability, depressed mood, problems of general psychosocial functioning and problems with occupation and activities were associated with longer duration of inpatient treatment. With respect to the migration-specific characteristics, immigrants with a shorter period of stay in Switzerland and the country of origin outside Europe spent less time in hospital. In detail, the length of inpatient stay of immigrants who lived in Switzerland for <5 years was by far the shortest and it gradually increased with the period of residence in the host country. Similarly, immigrants from a non-European country showed the shortest length of inpatient stay compared to immigrants from a Swiss neighbor or another European country. No statistically significant effect on the time spent in hospital was found for age, employment, legal residency status, language barrier and migration generation.

### Predictors of Length of Psychiatric Inpatient Stay

Results of a stepwise linear regression revealed (out of all sociodemographic and clinical patient characteristics considered in this study) a set of four predictor variables which explained length of inpatient stay best (Table 3). Results suggest that, even after controlling for the effects of behavioral and social problems (as assessed by the four HoNOS items included in the model), a “country of origin outside Europe” is a statistically significant predictor of a shorter inpatient stay. “Self-harm” and “substance use” were further factors predicting shorter length of stay, whereas the HoNOS rating “problems of general functioning” was significantly associated with a longer psychiatric inpatient episode. Other variables that showed significant effects in the univariate regression analysis did not further contribute to this model.

**Table 3:** Predictors of length of inpatient stay.

	**B**	**Beta**	**95% CI**	***P*-value**
**Migration-specific variables**				
Country of origin, non-European	−0.72	−0.26	−1.11; −0.33	<0.001
**Clinical variables**				
HoNOS 02, Self-harm	−0.14	−0.16	−0.27; −0.02	0.024
HoNOS 03, Substance use	−0.18	−0.25	−0.28; −0.08	<0.001
HoNOS 10, General functioning	0.17	0.21	0.06; 0.28	0.004

*Multiple regression analysis; forward variable selection; N = 176*.

*HoNOS, Health of the Nations Outcome Scales*.

*LOS, length of stay (log-transformed)*.

*Adjusted R^2^ = 0.208; F = 12.487 (4 df); P < 0.001*.

## Discussion

Shorter length of inpatient stay of foreign nationals in psychiatry (14) raises questions which are relevant for mental health care, namely of people who not rarely suffer from psychological stress and mental disorders. The purpose of this study was to evaluate which of the migration-specific, clinical and sociodemographic factors assessed at admission to the psychiatric hospital might contribute to an explanation of this shorter inpatient treatment.

The analysis of the immigrants' data revealed that a country of origin “outside Europe” was a strong predictor of a shorter length of psychiatric inpatient stay. This finding is in accordance with a previous Swiss case-control study that examined a variety of clinical measures, including the length of stay, in a large sample of immigrants (*N* = 4'826) compared to natives matched for age, gender and psychiatric diagnosis (10). This study showed that the length of psychiatric inpatient stay mainly depends on the country of origin, with immigrants from more distant countries, particularly from countries outside Europe, spending significantly less time in hospital compared to Swiss controls. On the other hand, immigrants from European countries, especially from countries located closest to Switzerland, showed a length of stay that was comparable to natives. Given the relatively small sample size in our study, we did not divide the group of immigrants into distinct geographical regions but assigned the country of origin to Swiss neighbor countries, other European and non-European countries. However, despite this rather rough classification, our results are still consistent to the study by Lay et al. (10) as those regions of origin that were reported to be associated with a shorter length of stay are predominantly located outside Europe.

Several explanations on the structural and individual level need to be discussed when interpreting the finding of a shorter length of stay of immigrants from countries outside Europe compared to Swiss neighbor and other European countries. First of all, there are no specific barriers for the access to and the period of inpatient psychiatric treatment as regards the health care policy in Switzerland, the cost recovery by the obligatory health insurance and the availability of sufficient financial and personnel resources in the health care system. It therefore appears less probable that structural conditions might be the reason for inequalities in inpatient treatment (19).

On the other hand, shorter length of stay of immigrants from non-European countries rather might be explained by cultural, ethnic or language differences. The immigrants' cultural and ethnic background was reported to have a strong influence on the vulnerability for mental illness as well as on resilience and coping mechanisms (3, 20). Moreover, it may also affect the patients' understanding of mental health care. In particular, the individual concepts of mental health and illness as well as the expectations and perceptions of psychiatric treatment show a large variety across different ethnicities (21–23). These cultural and ethnic differences are, in principal, more dominant in immigrants from more distant countries, especially from outside Europe, compared to immigrants from European countries that share, to some extent, a comparable cultural identity. In line with this, immigrants who lived for a shorter time in Switzerland spent shorter time in the hospital, compared to immigrants with a longer period of residence.

In connection with the cultural and ethnic differences, the dimensions of acculturation in the host country should also be taken into consideration when interpreting the results of this study. Berry (24) proposed the four dimensions of acculturation “integration,” “assimilation,” “separation,” and “marginalization” according to the psychological, behavioral and social changes resulting from being in continuous contact with other cultures. The acculturation domains “integration” and “assimilation” were often reported to be associated with better mental health status as well as higher rates of mental health care utilization, whereas “separation” and “marginalization” were linked to poor mental health outcomes and lower rates of mental health care utilization (25–28). The results of our study appear to confirm the impact of acculturation on the use of mental health care services. Immigrants with a shorter period of residence in Switzerland and a country of origin outside Europe, respectively, spent significantly shorter time in the hospital, presumably due to a lower degree of integration in the host country. On the other hand, it can be assumed that the longer length of inpatient stay of immigrants who lived in Switzerland for a longer time and/or came from a European country, and particularly from a Swiss neighbor country, resulted, at least in part, from a higher degree of integration. However, this conclusion should be drawn with caution as the dimensions of acculturation were not assessed in this study.

During inpatient treatment, language barriers may promote additional problems in the communication between immigrant patients and mental health professionals, increasing the risk of misunderstandings, misinterpretations and misdiagnosis that might result in earlier discharge of these patients (29). In our study, language barriers were not associated with the length of inpatient stay. That no such link could be found, however, is not surprising taking into consideration that the majority of this sample were second generation migrants, many of them speaking German as their native language.

Apart from the country of origin, specific behavioral and social problems, as assessed by the HoNOS, could be identified as predictors of the length of psychiatric inpatient treatment among immigrants. To the best of our knowledge, this is the first study that has analyzed the HoNOS in the context of migrant health. In particular, self-harm and substance use are associated with a shorter length of stay, whereas disturbances of general psychosocial functioning predicted a longer episode of inpatient treatment.

The HoNOS comprises a set of 12 single items with each of them representing a relatively independent scale. It was therefore recommended to consider the single items rather than the HoNOS total score for obtaining a differential and realistic picture of the impairment of patients with mental disorders in the clinical setting (16). Moreover, the use of the HoNOS items allows to assess specific psychopathological problems that are not fully covered by the principal psychiatric diagnoses. In accordance with this conclusion, we also found no association between the HoNOS total score and length of stay whereas some single HoNOS items contributed significantly to its prediction.

The results that have been obtained from the analysis of the HoNOS items largely confirm the findings of a previous study on the predictive value of psychopathological syndromes and symptoms for the length of stay. In this study, however, psychiatric inpatients in general, irrespective of the presence of a migration status, have been analyzed (30). The authors showed that crisis intervention, acute psychiatric care and compulsory admission as well as substance-related disorders were the strongest predictors of a shorter length of stay, whereas social withdrawal, depressiveness and memory disturbances increased the length of stay. The reasons for the necessity of crisis intervention, acute psychiatric care and compulsory admission were not given, but it can be assumed that these interventions were due to aggressive behavior, self-harm or suicidality. Taken together, it seems that the impact of psychopathological syndromes and symptoms on the length of inpatient stay is very similar in immigrants and non-immigrants. The present findings suggest that, beyond these common psychopathological factors, however, migration-related challenges moreover play a decisive role for treatment and discharge decisions in immigrants.

Several limitations have to be taken into account when interpreting the results of this study. (A) The present findings were not validated against a control group. It should be considered, however, that in this study we were interested in particular in migration-specific aspects which cannot be applied to non-immigrant patients. (B) Given the relatively small sample size, we did not perform a more fine-grained analysis of sample characteristics, such as country of origin. (C) The language barriers were estimated by the psychiatrists of the hospital according to the degree of difficulties in communication during inpatient treatment. These estimations strongly depend on the doctors' subjective perceptions. (D) We were not able to retrospectively assess the intercultural competences of the mental health care professionals who were involved in the inpatient treatment of the immigrants as well as in the decision about their discharge from the hospital. It can be suggested that poor intercultural competences might have contributed to differences in the length of inpatient stay, depending on the immigrants' cultural distance from the host country and their degree of integration. (E) Considering the overall small sample size of this study, the incomplete data, the interdependence of variables considered in the model as well as potential uncontrolled confounding, the results of the regression model should be regarded as preliminary unless this model is corroborated by further analyses. (F) Given the explorative nature of this study, further research in a larger sample of immigrants that are carefully matched to native patients is required to corroborate the findings and to better understand the specific needs of migrants with different cultural and ethnic background.

In summary, the results of this study show that shorter length of psychiatric inpatient stay among immigrants in Switzerland are predicted by their country of origin as well as by behavioral and social disturbances. The behavioral and social problems that were significantly associated with a shorter length of stay, however, do not appear to be migrant-specific, as similar findings have also been reported for non-immigrant patients. The shorter length of stay of immigrants from more distant countries, particularly from non-European countries, points to inequalities of inpatient psychiatric treatment. The consequences of such inequalities may comprise insufficient or inappropriate treatment, disappearance from the mental health care system, longer duration of untreated disease, higher rates of chronification and complications, and overall poorer outcome and prognosis. To eliminate disadvantages in mental health care, we must give more consideration to the cultural background and migrant history of immigrants in psychiatry.

## Data Availability Statement

The raw data supporting the conclusions of this article will be made available by the authors, without undue reservation.

## Ethics Statement

The studies involving human participants were reviewed and approved by Ethikkommission Nordwest- und Zentralschweiz (EKNZ). Written informed consent for participation was not required for this study in accordance with the national legislation and the institutional requirements.

## Author Contributions

PR, BH, ES, and WK contributed to the study conception and research design. RF and BL conducted the data collection and analysis. RF, BL, and PR contributed to the drafting of the manuscript. All authors commented and contributed to the final version of the manuscript and have given final approval.

## Conflict of Interest

The authors declare that the research was conducted in the absence of any commercial or financial relationships that could be construed as a potential conflict of interest.
