# Supplementary material for: Sociodemographic and Clinical Predictors of the Length of Psychiatric Inpatient Stay of Immigrants in Switzerland
Source: Front Psychiatry. 2020 Dec 9;11:585798. doi: 10.3389/fpsyt.2020.585798 (PMC7755930; doi:10.3389/fpsyt.2020.585798)
Supplement: Supplementary file 1 [file Table_1.docx]

**Supplement Table S1.** Number of cases, mean, standard deviation (SD), and median for length of stay (LOS; in days) across groups defined by HoNOS severity scores 0-4

HoNOS 1, Aggression and overactivity

| HoNOS  Severity | N | LOS  Mean | SD | Median |
| --- | --- | --- | --- | --- |
| 0 | 101 | 30.12 | 21.04 | 28.00 |
| 1 | 48 | 27.25 | 25.16 | 20.50 |
| 2 | 70 | 25.01 | 20.75 | 20.00 |
| 3 | 46 | 20.15 | 16.93 | 15.50 |
| 4 | 14 | 20.64 | 18.41 | 13.00 |
| Total | 279 | 26.23 | 21.19 | 21.00 |

HoNOS 2, Self-harm

| HoNOS  Severity | N | LOS  Mean | SD | Median |
| --- | --- | --- | --- | --- |
| 0 | 174 | 26.87 | 20.97 | 21.00 |
| 1 | 32 | 24.75 | 23.34 | 21.00 |
| 2 | 25 | 22.48 | 15.77 | 21.00 |
| 3 | 23 | 23.57 | 23.74 | 19.00 |
| 4 | 11 | 16.45 | 12.09 | 18.00 |
| Total | 265 | 25.48 | 20.81 | 20.00 |

HoNOS 3, Substance use

| HoNOS  Severity | N | LOS  Mean | SD | Median |
| --- | --- | --- | --- | --- |
| 0 | 118 | 30.83 | 22.00 | 27.00 |
| 1 | 31 | 30.39 | 23.47 | 28.00 |
| 2 | 35 | 19.69 | 17.20 | 15.00 |
| 3 | 49 | 19.82 | 16.14 | 18.00 |
| 4 | 36 | 21.08 | 17.47 | 17.50 |
| Total | 269 | 26.02 | 20.61 | 21.00 |

HoNOS 4, Cognition

| HoNOS  Severity | N | LOS  Mean | SD | Median |
| --- | --- | --- | --- | --- |
| 0 | 137 | 23.67 | 19.47 | 20.00 |
| 1 | 49 | 24.61 | 21.76 | 18.00 |
| 2 | 54 | 32.56 | 22.39 | 29.00 |
| 3 | 32 | 30.03 | 24.75 | 25.50 |
| 4 | 3 | 22.67 | 14.57 | 21.00 |
| Total | 275 | 26.31 | 21.27 | 21.00 |

HoNOS 5, Physical health

| HoNOS  Severity | N | LOS  Mean | SD | Median |
| --- | --- | --- | --- | --- |
| 0 | 167 | 23.81 | 21.43 | 19.00 |
| 1 | 29 | 30.52 | 23.09 | 27.00 |
| 2 | 38 | 29.03 | 18.99 | 24.50 |
| 3 | 29 | 28.97 | 17.97 | 28.00 |
| 4 | 14 | 29.57 | 23.36 | 20.50 |
| Total | 277 | 26.06 | 21.09 | 21.00 |

HoNOS 6, Hallucinations and delusions

| HoNOS  Severity | N | LOS  Mean | SD | Median |
| --- | --- | --- | --- | --- |
| 0 | 162 | 24.70 | 19.71 | 20.00 |
| 1 | 18 | 32.00 | 24.63 | 27.00 |
| 2 | 39 | 28.28 | 22.46 | 24.00 |
| 3 | 28 | 26.57 | 22.08 | 25.00 |
| 4 | 18 | 20.11 | 15.13 | 16.50 |
| Total | 265 | 25.61 | 20.49 | 20.00 |

HoNOS 7, Depression

| HoNOS  Severity | N | LOS  Mean | SD | Median |
| --- | --- | --- | --- | --- |
| 0 | 31 | 20.58 | 19.64 | 16.00 |
| 1 | 37 | 23.43 | 19.89 | 20.00 |
| 2 | 83 | 23.66 | 21.99 | 18.00 |
| 3 | 83 | 27.70 | 19.35 | 22.00 |
| 4 | 44 | 34.20 | 23.40 | 29.50 |
| Total | 278 | 26.16 | 21.20 | 21.00 |

HoNOS 8, Other symptoms

| HoNOS  Severity | N | LOS  Mean | SD | Median |
| --- | --- | --- | --- | --- |
| 0 | 66 | 24.11 | 16.45 | 20.00 |
| 1 | 9 | 41.89 | 35.17 | 26.00 |
| 2 | 58 | 23.53 | 19.70 | 19.50 |
| 3 | 89 | 26.29 | 21.78 | 21.00 |
| 4 | 34 | 35.44 | 24.99 | 33.00 |
| Total | 256 | 26.87 | 21.48 | 21.00 |

HoNOS 9, Social relations

| HoNOS  Severity | N | LOS  Mean | SD | Median |
| --- | --- | --- | --- | --- |
| 0 | 70 | 26.21 | 20.09 | 19.50 |
| 1 | 45 | 27.04 | 27.28 | 18.00 |
| 2 | 69 | 22.81 | 17.61 | 20.00 |
| 3 | 53 | 25.45 | 19.86 | 22.00 |
| 4 | 23 | 30.87 | 19.82 | 32.00 |
| Total | 260 | 25.71 | 20.81 | 20.50 |

HoNOS 10, General functioning

| HoNOS  Severity | N | LOS  Mean | SD | Median |
| --- | --- | --- | --- | --- |
| 0 | 71 | 17.56 | 17.29 | 11.00 |
| 1 | 37 | 20.62 | 16.58 | 15.00 |
| 2 | 74 | 28.65 | 20.55 | 26.00 |
| 3 | 59 | 31.29 | 22.53 | 29.00 |
| 4 | 28 | 35.46 | 23.73 | 29.50 |
| Total | 269 | 25.91 | 20.90 | 20.00 |

HoNOS 11, Housing

| HoNOS  Severity | N | LOS  Mean | SD | Median |
| --- | --- | --- | --- | --- |
| 0 | 116 | 24.92 | 21.02 | 20.00 |
| 1 | 42 | 24.50 | 19.87 | 20.50 |
| 2 | 42 | 25.17 | 20.81 | 20.00 |
| 3 | 39 | 27.74 | 23.94 | 22.00 |
| 4 | 17 | 32.06 | 20.48 | 38.00 |
| Total | 256 | 25.80 | 21.16 | 20.00 |

HoNOS 12, Activities

| HoNOS  Severity | N | LOS  Mean | SD | Median |
| --- | --- | --- | --- | --- |
| 0 | 67 | 24.85 | 21.67 | 20.00 |
| 1 | 39 | 17.18 | 14.46 | 15.00 |
| 2 | 54 | 25.09 | 18.57 | 23.50 |
| 3 | 63 | 28.49 | 22.77 | 21.00 |
| 4 | 33 | 32.58 | 24.37 | 31.00 |
| Total | 256 | 25.63 | 21.08 | 20.00 |
